# A Narrative Review of Thromboprophylaxis in Obese Patients Undergoing Surgery: What Do You Need to Know?

**DOI:** 10.7759/cureus.94258

**Published:** 2025-10-10

**Authors:** Luis Carreto, Maria Garcia

**Affiliations:** 1 Anesthesiology, Hospital Ángeles Tijuana, Tijuana, MEX; 2 Anesthesiology, Hospital General de Tijuana, Tijuana, MEX

**Keywords:** bariatric, obese patient, surgery, thromboembolic event, thromboprophylaxis

## Abstract

Surgeries in obese patients have increased significantly, leading to a rise in perioperative complications such as embolic events. Preventing these complications in obese patients presents pharmacological challenges and involves optimization of guidelines and follow-up considerations, as well as the need for sufficient evidence to ensure safety with minimal risk. In this literature review, we address aspects of dosing with unfractionated and low-molecular-weight heparins, the role of factor Xa inhibitors, and the initiation and duration of thromboprophylaxis in obese patients undergoing surgery. We also review the current role of inferior vena cava filters as a preventive measure.

## Introduction and background

Obesity, the excessive accumulation of fat, is commonly defined as a body mass index (BMI) of ≥ 30 kg/m², and morbid obesity is commonly defined as a BMI of ≥ 40 kg/m² [1]. In 2022, the World Health Organization estimated that the global prevalence of obesity had more than doubled since 1990, with more than 2.5 billion adults being classified as obese or overweight [1]. Likewise, surgical interventions for obesity have increased fivefold worldwide over the past 15 years, with variation across countries, and, over only 10 years (2008-2018), 6.5 million people underwent bariatric surgery [2].

Obesity is recognized as a prothrombotic state characterized by multiple interrelated pathophysiological mechanisms, including impaired fibrinolysis, chronic inflammation, endothelial dysfunction, elevated levels of procoagulant factors, and platelet activation [3]. Adipose tissue releases adipocytokines and free fatty acids that promote inflammation, leukocyte recruitment, platelet aggregation, and endothelial dysfunction, and these changes double the risk of venous thromboembolism (VTE), including deep vein thrombosis (DVT) and pulmonary embolism (PE), compared with individuals who have BMIs in the normal range [3].

Meta-analyses and epidemiological studies have identified a linear relationship between BMI and VTE risk, with a nearly six-fold increase in the risk for individuals who have a BMI of ≥ 35 kg/m² [3]. Despite this increased risk, the clinical management of anticoagulation in obese patients presents significant challenges. The dosing and monitoring of anticoagulants often lack guidance based on high-quality studies because obese patients, particularly those with morbid obesity, are underrepresented or excluded from clinical trials of anticoagulants [3].

As a result, there is a notable lack of consistency in the prophylaxis and treatment of VTE in this population, with significant variations in dosing strategies, monitoring, and the choice of anticoagulants. The aim of this narrative review is to summarize the current evidence regarding the optimization of thromboprophylaxis in obese patients by addressing risk classification, the use of mechanical devices and pharmacological agents, dosing, and duration. We also analyze the benefits and disadvantages of the various preventive interventions.

## Review

Methodology

This article presents a literature review that synthesizes data from meta-analyses, retrospective studies, cohort studies, case series, and clinical guidelines related to perioperative thromboprophylaxis in obese patients. The studies included non-pregnant adult patients, with a maximum publication window of 10 years (from 2015 to 2025).

We consulted MEDLINE, Embase, and Scopus databases through PubMed for the information search using the Medical Subject Headings (MeSH) terminology and Boolean operators: “thromboprophylaxis AND obesity”, “thromboembolic risk”, “thromboprophylaxis AND bariatric surgery”, “anticoagulants AND obesity”, and “venous thromboembolism OR bariatric surgery”.

Mechanical thromboprophylaxis

A retrospective review of patients who underwent general and bariatric surgery found that, among 65 patients who underwent 76 procedures and had a mean age of 51 years and an average BMI of 45, 2.6% developed postoperative VTE when intermittent pneumatic compression stockings and pharmacological prophylaxis were used [4]. The use of pneumatic compression stockings and early ambulation is described in the European guidelines as effective measures for reducing VTE [5]. Similarly, a 2018 Cochrane Systematic Review concluded, with a moderate to high level of evidence, that the combination of intermittent pneumatic compression stockings and pharmacological prophylaxis reduced the incidence of embolic events [6].

Pharmacological thromboprophylaxis

Fixed Doses and Weight-Adjusted Regimens

Obesity, particularly class ≥ 2 obesity, can alter the pharmacokinetics (PK) of medications, leading to inadequate dosing when fixed doses or weight-adjusted regimens are used. In obese patients, gastric emptying is accelerated, and the absorption and bioavailability of some oral drugs may be reduced. More specifically, the lean mass/fat ratio correlates with the volume of distribution (Vd) of lipophilic drugs. For hydrophilic drugs, such as low-molecular-weight heparin (LMWH), the Vd increases nonlinearly with body weight, so overdosing may occur in morbidly obese patients. In addition, the lipophilic properties of some drugs further affect their PK, while hepatic biotransformation through certain cytochrome P450 enzymes may be reduced [7,8].

**Figure 1:**
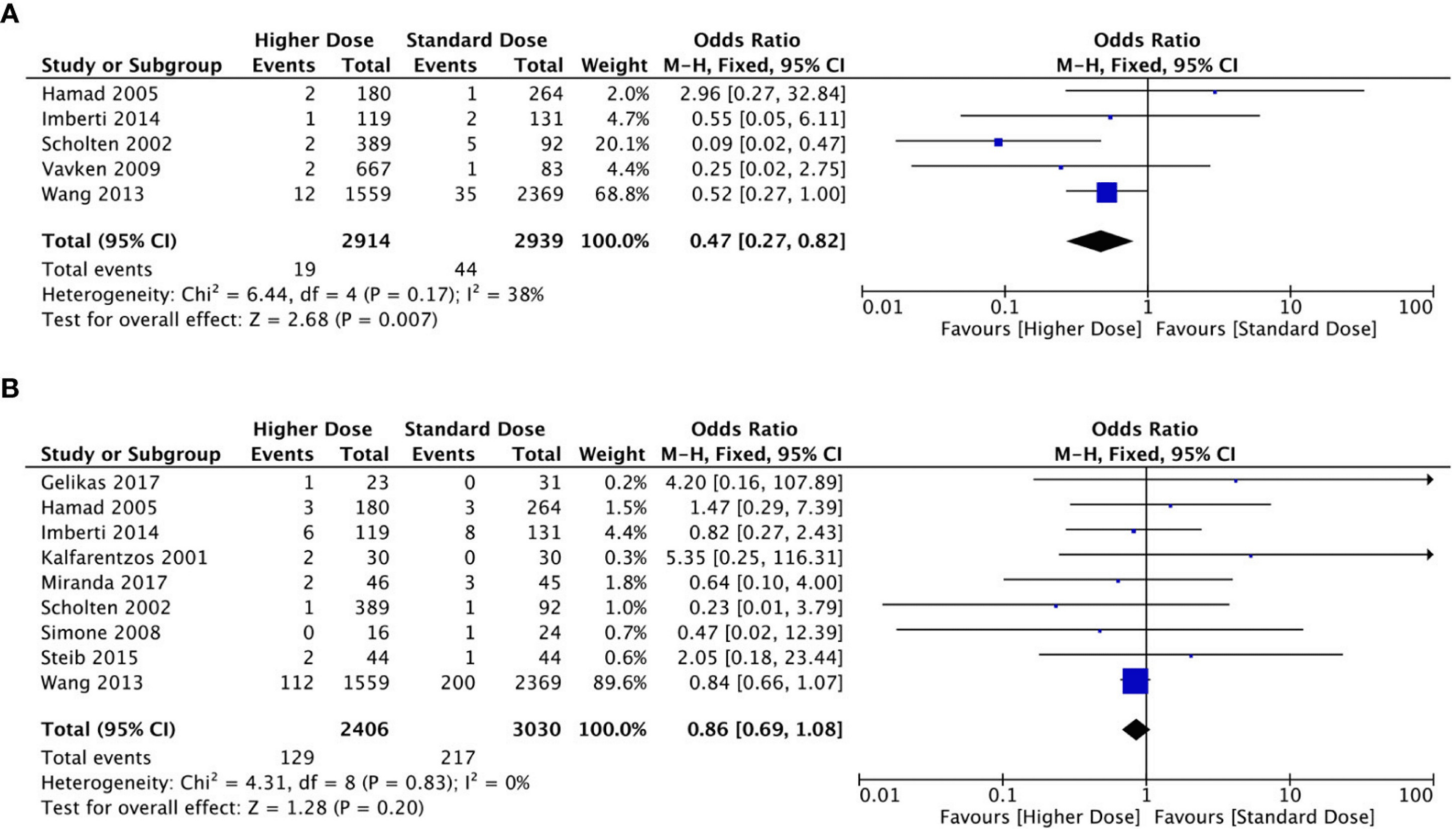
Forest plots comparing high-dose with standard-dose LMWH for VTE prophylaxis in obese patients. (A) VTE occurrence. (B) Bleeding events. LMWH: low-molecular-weight heparin; VTE: venous thromboembolism; CI = confidence interval; df = degrees of freedom. Figure reproduced from Liu et al. [9] under the terms of the Creative Commons Attribution License (CC BY). Study references are included in the Appendix.

Since LMWH is administered subcutaneously, it has been hypothesized that its absorption may be prolonged in obese patients. However, if the Vd is limited to the intravascular compartment, dosing based on total body weight may lead to supratherapeutic dosing and an increased risk of bleeding. A recent systematic review of 17 studies involving 2,900 patients showed a trend favoring the use of higher prophylactic anticoagulant doses in those with BMIs of > 30 to reduce the recurrence of embolic events without significant bleeding effects [9], as shown in Figure 1.

On the other hand, a systematic review of 11 studies involving more than 13,000 patients found that neither benefits nor risks were associated with weight-adjusted dosing in patients with BMIs of < 40 [10].

A multivariate analysis of adjustments to LMWH dosing according to total body weight failed to demonstrate a greater benefit in preventing embolic events compared with fixed or non-weight-adjusted doses, at least in patients with class 2 obesity or weighing < 121 kg [11]. Martin et al. conducted a retrospective study to identify the optimal dose of enoxaparin for VTE prevention in obese patients (with BMIs ranging from 30 to 50) based on the therapeutic levels of anti-factor Xa (AFXa) antibodies and found a subcutaneous dose of 40 mg every 12 hours to be safe and effective [12]. In a meta-analysis, Amaral et al. reported varying the doses of LMWH according to weight: patients with BMIs of < 50 received 40 mg of subcutaneous enoxaparin every 12 hours, and all of the patients with BMIs of > 50 who received a total of 60 mg enoxaparin underwent bariatric surgery without evidence of thromboembolic complications or postoperative bleeding [13].

Recent consensus supports the use of increased prophylactic doses in patients with class 2 obesity or greater to reduce the incidence and recurrence of VTE and achieve therapeutic AFXa levels [14]. Heparin has a narrow therapeutic window and a nonlinear pharmacokinetic profile that make it challenging to dose, but prophylactic VTE dosing for heparin is fixed and typically administered subcutaneously in doses of 5,000 units every eight hours [14]. Regis et al. evaluated the safety of high prophylactic doses compared with standard prophylactic doses of unfractionated heparin (UFH), specifically, 7,500 units every eight hours compared with 5,000 units every eight hours­ in 320 obese patients, and found a significant difference in the incidence of bleeding between the two groups (p = 0.008) but no significant difference in the incidence of VTE [15]. A multivariate retrospective analysis showed that the use of high, weight-adjusted doses of UFH was an independent predictor of bleeding compared with LMWH in morbidly obese patients, but no significant difference in the incidence of VTE was observed [16].

The safety and cost of thromboprophylaxis with enoxaparin or UFH in inpatients with obesity were evaluated in a retrospective cohort of 61,793 inpatients, 66% of whom received enoxaparin and 34% of whom received UFH [17]. The researchers found that the use of enoxaparin was associated with decreases in the adjusted odds of VTE, PE-related mortality, in-hospital mortality, major bleeding, and significantly lower total hospitalization costs compared with the use of UFH (p < 0.002 in each case).

Doses in obese patients with renal dysfunction

Lee et al. studied 99 morbidly obese patients with renal dysfunction [18]. Those with creatinine clearance (CrCl) > 30 ml/min received enoxaparin at a dose of 1 mg/kg every 12 hours, and those with CrCl < 30 ml/min received 1 mg/kg every 24 hours. These researchers found that 50% of all patients had supratherapeutic anti-Xa levels and suggested that monitoring these levels is necessary to ensure the safe and effective use of enoxaparin in this cohort.

Lalama et al. used their own protocol with reduced enoxaparin doses: patients with CrCl ≥ 30 ml/min received 0.75 mg/kg every 12 hours, and those with CrCl < 30 ml/min received 0.75 mg/kg every 24 hours [19]. These researchers reported that 77% of the patients showed anti-factor Xa activity within the therapeutic ranges and that no cases of bleeding or embolic events were observed. The routine monitoring of AFXa levels is not recommended except in contexts, such as advanced age or renal impairment (creatinine clearance < 30 ml/min), in which drug metabolism may be altered and lead to non-therapeutic anticoagulant levels, thus exposing patients to the risk of bleeding or thrombosis [20].

Factor Xa inhibitors (xabans)

Some data suggest that, in the immediate postoperative period, the concentrations of xabans (rivaroxaban, apixaban, and dabigatran) may be affected by malabsorption, though the impact of these anticoagulants on the effectiveness of prophylactic treatments has not been clearly established. Model-based pharmacokinetic simulations, mainly from randomized studies that reported anti-Xa levels after the use of xabans, did not show significant pharmacological changes in patients with class 2-3 obesity [7].

A recent systematic review and meta-analysis of eight studies involving more than 30,000 patients demonstrated that the efficacy of xabans is similar to that of warfarin, with a lower likelihood of major bleeding [21]. Elshafei et al. confirmed these findings in another systematic review and meta-analysis [22]. Further, in a comparative study of warfarin, rivaroxaban, and apixaban, the latter anticoagulant was associated with fewer VTE and bleeding events than warfarin [23].

A post hoc analysis found that apixaban and enoxaparin were equally effective and safe in preventing PE and VTE in obese and non-obese patients, but the incidence of bleeding was significantly lower with apixaban [24]. A systematic review and meta-analysis of five studies involving more than 6,000 patients found that xabans and LMWH were equally effective and safe [25].

In sum, xabans can be used at standardized doses in obese patients regardless of BMI, with a favorable safety profile, and represent an alternative to vitamin K antagonists.

Initiation and duration of thromboprophylaxis

According to the Enhanced Recovery After Surgery in Bariatric Surgery (ERABS) protocol, the duration of thromboprophylaxis should be individualized according to thrombotic risks [26]. The Enoxaparin Fondaparinux for Thromboprophylaxis (EFFORT) trial compared fondaparinux (a direct AFXa inhibitor) administered postoperatively at 5 mg subcutaneously with enoxaparin (an LMWH) administered pre- and postoperatively at a dose of 40 mg subcutaneously twice daily as a prophylactic strategy for reducing the incidence of VTE in patients undergoing laparoscopic bariatric surgery [27]. The average length of stay in this study was 2.5 days. The findings indicated that, although fondaparinux was more effective in maintaining the recommended activity level of AFXa, both regimens were effective and associated with insignificant rates of DVT (2.4% and 2.2%, respectively; p = 1.00).

A 2022 Cochrane Systematic Review that included seven randomized controlled trials involving 1,045 bariatric surgery patients found little to no difference between starting prophylaxis with heparin 12 hours before surgery and starting prophylaxis after surgery in terms of the risk of VTE, DVT, bleeding, or related mortality [13]. A study involving a cohort of more than 3,000 patients found that pharmacological and mechanical thromboprophylaxis, in addition to early mobilization during hospital stays, was as effective as a 15-day regimen in preventing VTE. However, this was a single-center study with unequal group sizes that included the use of tranexamic acid [28]. A 2017 study showed a tendency toward thrombosis in morbidly obese patients within two weeks postoperatively [29]. A 2024 multivariate analysis by Ali et al. found that bariatric surgery patients with a BMI of > 40 and additional thromboembolic risk factors, such as chronic obstructive pulmonary disease, obstructive sleep apnea, pre-existing VTE, hospital stays of > three days, and glycated hemoglobin (HbA1c) > 7.1, may benefit from extended thromboprophylaxis [30].

By contrast, a multiple regression study by Aminian et al. involving 91,963 bariatric surgery patients from the American College of Surgeons-National Surgical Quality Improvement Program (ACS-NSQIP) database found that more than 80% of VTE cases occurred within the first 30 postoperative days [31]. These researchers identified 10 predictors: congestive heart failure, paraplegia, reoperation, resting dyspnea, age of > 60 years, BMI of > 50, surgeries other than gastric band, male sex, operative time of > three hours, and hospital stays of > three days. A validated model was created to calculate the postoperative VTE risk by considering the types of surgery. Aminian et al. accordingly recommended extended thromboprophylaxis for patients at high risk.

Abuoglu et al. conducted a study of 368 patients who received LMWH (0.2 ml nadroparin) 12 hours before surgery and resumed 24 hours postoperatively (0.4 ml nadroparin) for up to 15 days to determine the efficacy of LMWH in preventing VTE in bariatric surgery patients and reported 0% DVT [32]. Another study, with a cohort of more than 110,000 bariatric surgery patients who received a 90-day post-discharge follow-up, found that VTE was strongly associated with individual risk factors such as a history of VTE, open surgery, and heart failure [33]. The findings indicated that extended pharmacological prophylaxis (15-30 days postoperatively) reduced both the risk of VTE and the readmission rate compared with patients who did not receive such prophylaxis. Therefore, in patients with thrombotic risk factors in addition to obesity and more than 24 hours of exposure, it may be advisable to extend thromboprophylaxis to two weeks postoperatively.

Monitoring AFXa activity

LMWH inhibits factor Xa by binding to antithrombin, so its activity can be monitored using serum AFXa levels rather than the activated partial thromboplastin time. In one study, the peak AFXa level was reached three to five hours after administration of the third dose. The target AFXa levels have been relatively well defined for therapeutic LMWH doses but not for prophylactic doses, especially in obese patients, and this study suggested that 0.2-0.5 IU/mL is a reasonable target range of AFXa for LMWH prophylaxis of VTE [34].

However, AFXa levels lack standardization and reproducibility and are weakly associated with bleeding and thrombotic states [14,35]. A prospective study involving 236 patients reported that AFXa measurements helped establish the optimal thromboprophylactic state in subjects weighing > 150 kg or with BMIs of > 40 [21]. Routine monitoring of AFXa levels is not recommended, except in contexts such as advanced age or renal dysfunction [20].

Inferior vena cava filters

Since there have been no well-designed prospective studies or randomized controlled trials of the effectiveness of the preoperative insertion of inferior vena cava filters (IVCFs), their use for primary thromboprophylaxis remains controversial. The Society of Interventional Radiology and the American College of Radiology provide limited support for this intervention, whereas the American College of Chest Physicians opposes its use.

**Figure 2:**
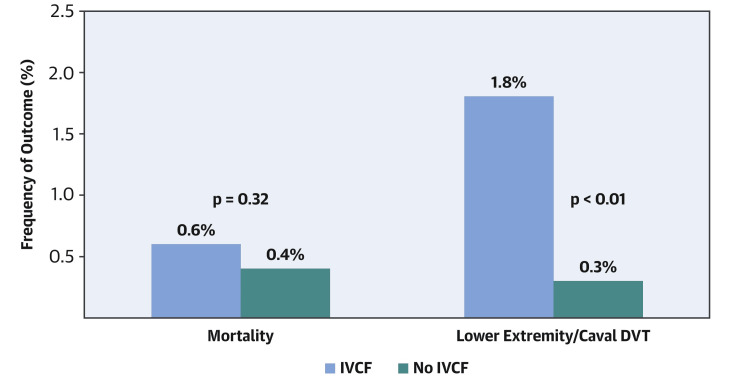
Clinical outcomes of the prophylactic use of IVCFs before bariatric surgery. IVCF: inferior vena cava filter; DVT: deep vein thrombosis. Figure reproduced with permission from Reddy et al. [36].

A prospective observational study over 10 years involving more than 200,000 patients found that only 1,041 of them underwent insertion of an IVCF before bariatric surgery [36]. In this study, compared with the patients who did not receive a filter, the patients who did receive a filter had a threefold higher risk of death and pulmonary embolism (Figure 2).

A 2015 systematic meta-analysis by Rowland et al. of the prophylactic use of IVCFs in bariatric surgery patients included 18 studies [37]. In these studies, filters were placed preoperatively in obese patients at high risk of VTE. The findings indicated that DVT was 20% more frequent, and pulmonary embolism was 6% more frequent, among the patients with IVCFs than those without, thus demonstrating that the filters did not reduce the incidence of VTE.

The heterogeneous nature of the evidence regarding the safety and efficacy of IVCFs in obese surgical patients complicates recommendations for the use of these filters.

Thromboprophylaxis in obese patients, surgery, and travel

The association between prolonged travel and VTE was first documented in the 1950s and continues to be observed. A recent systematic review of 18 studies analyzed the relationship between long-distance travel and VTE and found a low to moderate association owing to the quality of the available evidence [38]. The researchers reported a 26% increase in the risk of VTE for every two hours of air travel, starting from a cutoff point of four hours, and that compression stockings were effective in preventing VTE.

A meta-analysis of seven retrospective studies involving 24,975 patients estimated the postoperative VTE risk associated with recent air travel, with subgroup analyses including pre- and postoperative flights, flights longer than four hours, and high-risk surgeries for embolic events [39]. These researchers found that the risk of VTE was 7.86% (95% CI: 0.23-265.26) for the pre- and postoperative flight group and 1.31% (95% CI: 0.63-2.71) for postoperative flights only, 2.35% (95% CI: 0.29-19.36) for air travel of greater than hours, and 1.20% (95% CI: 0.45-3.20) for high-embolic-risk surgeries combined with air travel.

The current evidence indicates that air travel does not confer an additional VTE risk in patients who have undergone surgery. However, this conclusion does not take into account confounding factors, and, given the low quality of evidence in most of these studies, no specific preventive intervention can be recommended for these situations.

## Conclusions

The aim of this review was to address key questions regarding the perioperative management of thromboprophylaxis in obese patients. Heparin dosing is usually modified in cases of severe obesity, and the duration of prophylaxis may need adjustment when additional thromboembolic risk factors are present in obese surgical patients. Factor Xa inhibitors (xabans) are a viable option with a good safety and efficacy profile for prevention in this population, whereas monitoring AFXa activity should be reserved for special situations. There is insufficient evidence either to recommend the use of IVCFs or to issue specific guidance for patients who undergo surgery and need to travel immediately afterward.

## Appendices

**Table 1:** Studies cited in Figure 1.

Authors	Article title	Year of publication	Volume	Page range	DOI
Hamad GG, Choban PS	Enoxaparin for thromboprophylaxis in morbidly obese patients undergoing bariatric surgery: findings of the prophylaxis against VTE outcomes in bariatric surgery patients receiving enoxaparin (PROBE) study	2005	15	1368-74	10.1381/096089205774859245
Imberti D, Baldini E, Pierfranceschi MG, et al.	Prophylaxis of venous thromboembolism with low molecular weight heparin in bariatric surgery: a prospective, randomised pilot study evaluating two doses of parnaparin (BAFLUX study)	2014	24	284-91	10.1007/s11695-013-1105-X
Scholten DJ, Hoedema RM, Scholten SE	A comparison of two different prophylactic dose regimens of low molecular weight heparin in bariatric surgery	2002	12	19-24	10.1381/096089202321144522
Vavken P, Lunzer A, Grohs J	A prospective cohort study on the effectiveness of 3500 IU versus 5000 IU bemiparin in the prophylaxis of postoperative thrombotic events in obese patients undergoing orthopedic surgery	2009	121	454-8	10.1007/s00508-009-1175-x
Wang TF, Milligan PE, Wong CA, Deal EN, Thoelke MS, Gage BF	Efficacy and safety of high-dose thromboprophylaxis in morbidly obese inpatients	2013	111	88-93	10.1160/TH13-01-0042
Gelikas S, Eldar SM, Lahat G	Anti-factor Xa levels in patients undergoing laparoscopic sleeve gastrectomy: 2 different dosing regimens of enoxaparin	2017	13	1753-9	10.1016/j.soard.2017.07.027
Kalfarentzos F, Stavropoulou F, Yarmenitis S, Kehagias I, Karamesini M, Dimitrakopoulos A, Maniati A	Prophylaxis of venous thromboembolism using two different doses of low-molecular-weight heparin (nadroparin) in bariatric surgery: a prospective randomized trial	2001	11	670-6	10.1381/09608920160558588
Miranda S, Le Cam-Duchez V, Benichou J, Donnadieu N, Barbay V, Le Besnerais M, Delmas FX, Cuvelier A, Lévesque H, Benhamou Y, Armengol G	Adjusted value of thromboprophylaxis in hospitalized obese patients: A comparative study of two regimens of enoxaparin: the ITOHENOX study	2017	155	1-5	10.1016/j.thromres.2017.04.011
Simone EP, Madan AK, Tichansky DS, Kuhl DA, Lee MD	Comparison of two low-molecular-weight heparin dosing regimens for patients undergoing laparoscopic bariatric surgery	2008	22	2392-5	10.1007/s00464-008-9997-6
Steib A, Degirmenci SE, Junke E, Asehnoune K, Figier M, Pericard C, Rohr S, Letessier E, Brunaud L, Vix M, Zobairi F, Grunebaum L, Toti F	Once versus twice daily injection of enoxaparin for thromboprophylaxis in bariatric surgery: effects on antifactor Xa activity and procoagulant microparticles. A randomized controlled study	2015	12	613-21	10.1016/j.soard.2015.08.505
